# Gender differences in emotionality and sociability in children with autism spectrum disorders

**DOI:** 10.1186/2040-2392-5-19

**Published:** 2014-02-28

**Authors:** Alexandra M Head, Jane A McGillivray, Mark A Stokes

**Affiliations:** 1 School of Psychology, Deakin University, 221 Burwood Highway, Burwood, VIC 3125, Australia

**Keywords:** Autism, Female profile, Friendship, Social skills

## Abstract

**Background:**

Four times as many males are diagnosed with high functioning autism compared to females. A growing body of research that focused on females with autism spectrum disorder (ASD) questions the assumption of gender invariance in ASD. Clinical observations suggest that females with ASD superficially demonstrate better social and emotional skills than males with ASD, which may camouflage other diagnostic features. This may explain the under-diagnosis of females with ASD.

**Methods:**

We hypothesised that females with ASD would display better social skills than males with ASD on a test of friendship and social function. One hundred and one 10- to 16-year-olds (ASD females, n = 25; typically developing (TD) females, n = 25; ASD males, n = 25; TD males, n = 26) were interviewed (using the friendship questionnaire (FQ)) with high scores indicating the child has close, empathetic and supportive relationships. One parent of each child completed the FQ to assess whether there are differences in perception of friendships between parents and children.

**Results:**

It was found that, independent of diagnosis, females demonstrated higher scores on the FQ than males. Further, regardless of gender, children with ASD demonstrated lower scores than TD children. Moreover, the effect of ASD was independent of gender. Interestingly, females with ASD and TD males displayed similar scores on the FQ.

**Conclusions:**

This finding is supported by clinical reports that females with ASD have more developed social skills than males with ASD. Further research is now required to examine the underlying causes for this phenomenon in order to develop gender-appropriate diagnostic criteria and interventions for ASD.

## Background

In recent years, there has been a considerable body of research exploring the diagnostic criteria and differing behavioural expressions of autism spectrum disorders (ASDs). Most of this research has been undertaken focusing on males with high functioning autism or Aspergers Syndrome (hereafter referred to as ASD). However, research into the female phenotype has been limited and findings have generally been inconsistent [1-3]. Within this limited literature, it has been suggested that, in order for females to be diagnosed with ASD, they will generally be more impaired than males and frequently have an accompanying intellectual disability [1,3,4].

It is generally accepted that, for every female diagnosed with ASD, there are at least four males diagnosed [5], whereas for those who are also profoundly intellectually disabled the ratio is closer to two males for each female [6]. Among high functioning males and females, there is a common belief that males and females do not differ behaviourally, emotionally or intellectually [7]. However, a fundamental flaw exists in this argument. The supposed gender invariance is not consistent with gender differences evident in the typically developing (TD) population, such as superior social skills observed in females or better friendship stability seen in young males [8]. Furthermore, gender has been shown to impact greatly on many major aspects of development; for instance, through childhood, males demonstrate superior motor skills while female children demonstrate more advanced interpersonal and emotional skills [8]. As such, it seems implausible that gender would not have a significant impact on the expression of ASD. Whether these differences in gender are supported in the ASD population is yet to be examined.

The difficulty in identifying females with ASD may be further compounded by gender differences in sociability, friendships and emotionality in the TD population. That is, social-communicative difficulties are key diagnostic criteria for ASD; yet females with ASD, like TD females, may be less impaired (or demonstrate strengths) in these areas compared to males with ASD [9]. This suggestion, however, has yet to be fully tested. It would appear that the nature of social-communicative and friendship differences among TD females and males very likely impacts differences among males and females with ASD, and it would be useful to assay this empirically, in turn possibly leading to the development of more specific and sensitive diagnostic criteria that have regard to gender. This is critically important as the accurate diagnosis of females with ASD may enhance their access to early and ongoing intervention and support to maximise their development and wellbeing. Findings may also inform specifically tailored intervention programs for females with ASD in a range of areas, including developing and maintaining friendships.

Friendship is widely pronounced to be fundamentally based on three tenants of companionship: intimacy, trust and affection [10-14]. Friendships are integral for developing social supports, essential social skills and gender roles [11,15,16]. Intimate friendships among TD males are characterised by rough and tumble play, minimal close connection and a large group of like-minded friends, acquaintances, playmates or strangers [11,16-19]. TD males are more likely to be inclusive in their networks compared to TD females but are more prone to conflict [12,20]. In contrast, intimate friendships involving TD females are characterised by sharing and closeness, based on mutual interest, reciprocity and affection. Females also demonstrate greater skills in socio-cognitive functioning compared to males [21]. They tend to have two or three close friends as well as a wider group of friends with whom they regularly interact [16,20-23]. Interestingly, friendship stability in females tends to be lower than in males [11], as females are inclined to change friends or terminate friendships more readily than their male counterparts [19]. It is generally accepted that TD females demonstrate superior social skills compared to TD males [24].

Friendships within the ASD population, however, are characterised somewhat differently. For example, it is well documented that children and adults with ASD regularly report difficulty with associating with peers and maintaining friendships [25,26]. Using 60 children with ASD, Kasari and colleagues examined social relationships and report that they are more likely to operate on the fringe of their friendship circles and report lower quality of friendships [27]. Children with ASD are reported to be at greater risk of experiencing rejection than TD children. Specifically, the social networks of 44 children with ASD and other disabilities were assessed and the authors concluded that children with ASD are more likely to be rejected than otherwise disabled peers by their TD peers [28]. Reports of feeling abandoned or isolated are not uncommon from children with ASD and this is probably due to their trouble interpreting body language and other social cues [29]. Koning and Magill-Evans [30] found support for this pattern when they investigated social perception and social networks among 42 boys with and without ASD. Carrington and colleagues [31] interviewed five children with ASD and established common barriers for children with ASD to develop and maintain friendships. Specifically, they noted that constantly talking about special interests or not wanting to venture outside of comfort zones such as going to new places, or playing a new game are also commonly described as barriers to maintaining friendships for people with ASD [31,32]. Daniel and Billingsley [33], based on interviews with seven boys with ASD, report that males with ASD often focus their friendships on mutual interests and activities but noted that, in the absence of these shared interests, the friendships rarely continue. This behaviour is consistent with TD males, whereby males are more likely to socialise around a common interest such as a particular sport or game rather than socio-emotional activities [34].

Currently, the literature in this area largely focuses on males with ASD, whereby females with ASD are represented by only a few participants in the sample or are excluded altogether [1]. As such, it is difficult to gain a clear understanding of friendships and sociability for females on the autism spectrum. Due to this limitation in the literature, it may be more useful to utilise clinical accounts of the different presentations of females and males with ASD to better understand how these two groups develop and maintain friendships [35-38].

In a clinical description, Attwood [9] describes females who develop coping mechanisms or an ability to camouflage their social inadequacies through imitating and memorising acceptable social behaviours. This clinical description supports the Camouflage Hypothesis, originally proposed by Wing [39]. Wing proposed that females with ASD develop social skills and coping mechanisms that allow them to blend in or camouflage themselves into society, obscuring a likely diagnosis of ASD, even though there may be other indications of the condition. Specifically, while these skills are not representative of TD females, they may be superior to those characteristically expressed by a male with autism. As such, females on the spectrum still exhibit social deficits compared to TD females, but display relative strengths in this area compared to males on the spectrum. Wing’s hypothesis suggests that females with ASD adapt to and imitate appropriate social skills, allowing them to assimilate into neurotypical social circles. Attwood [9] reports that clinically he finds females with ASD utilise cognitive skills to respond to social situations. Similarly, Kopp and Gillberg [37] describe cases of females who clearly demonstrate autistic-like behaviours but did not fully meet criteria for ASD or Aspergers Syndrome, as specified at that time (by the Diagnostic and Statistical Manual of Mental Disorders Third Edition revised). These females are reported to be a diagnostic anomaly whereby their social profile was not at all similar to traditional descriptions of autistic social behaviour [37].

The under-diagnosis of females with ASD has been examined in three more recent studies. Using autism criteria on the Childhood Autism Spectrum Test, Dworzynski (2012) compared males and females aged between 10 and 12 years who were drawn from more than 15,000 sets of twins in the United Kingdom [6]. When intelligence quotient (IQ) and functioning levels were equivalently high, females were significantly less likely to be diagnosed with an ASD than males. Furthermore, the females displayed fewer socio-communicative symptoms than the males. A similar pattern was reported by Lai and colleagues [40] with 83 age- and IQ-matched adult males and females with ASD. Specifically, females demonstrated fewer socio-communicative deficits than males and they were less likely to be diagnosed [41]. In contrast to these studies, Carter and colleagues (2007) found no differences between males and females with ASD on measures of social functioning, or on cognitive and developmental functioning. It is important to note that this study was conducted with 100 children (22 females) who had a mean age of 28 months. Comparison of these studies is difficult as their respective age ranges are from babies to adults, thus representing an array of developmental abilities and stages.

Baron-Cohen and Wheelwright [42] also examined the possibility that females with ASD exhibit social skills which are closer in quality and content to that of TD females than males with ASD. They assessed 27 TD adult males and 49 TD adult females, and compared these to 51 adult males and 17 adult females diagnosed with ASD on the friendship questionnaire (FQ) [40]. As expected, females scored higher on the FQ than males, regardless of diagnosis. Moreover, TD individuals scored higher than those with ASD regardless of gender and no significant differences were found between the males and the females with ASD.

Although these findings are consistent with current clinical observations of gender differences in sociability and emotionality in TD and ASD individuals, caution should be exercised when generalising these findings to the ASD population. The authors did not triangulate their measurements, such as with reports from parents or friends. This is of concern, given the indication that individuals with ASD tend to overestimate the significance of their friendships and relationships [43]. In addition, the age range of the participants in the study was broad (14 to 64 years). Such a large age range poses a number of potential issues. For example, 14-year-olds and 64-year-olds are unlikely to share the same relationship dynamics; their life stage is critical to the development of social skills and friendships [23]. As Baron-Cohen and Wheelwright did not match subjects, differences in ages across the groups would have impacted upon group means on the FQ, possibly enhancing or obscuring group differences in unknown manners. Most importantly, though, differences in ages within each sample would increase error variance of the FQ scores, mathematically rendering a significant result considerably less likely to be obtained. A considerable improvement would be to restrict the age range, for instance to adolescents. In turn, this would provide insight into the social and empathic skills of individuals where these skills are still developing and are most likely to be referred for diagnosis and subsequent intervention. Finally, as with the majority of studies examining ASD, the ratio of male to female participants was not equal. Any heterogeneity of variance across gender therefore renders group differences even more difficult to recognise. Consequently, conclusions made in regard to females with ASD must be taken with caution.

The aim of the present study was to examine the female presentation of ASD by exploring the social aspects of children with ASD. If social and emotional advantages possessed by TD females compared to TD males are in anyway evident in females with ASD, as the evidence above would suggest, differences should be evident in the way females with ASD behave and appear compared to males with ASD. In conjunction with Wing’s Camouflage Hypothesis, it was expected that females with ASD would score differently to males with ASD on Baron-Cohen and Wheelwright’s FQ [42]. More specifically, it was expected that females with ASD would score higher than males with ASD on the FQ. It was also expected that, regardless of diagnosis, female participants would score higher than male participants. As it has been well established that people with ASD experience social deficits, it was also anticipated that TD participants would score higher than ASD participants, regardless of gender.

## Method

### Ethics approval

Participation was voluntary and informed consent was given by all parents and children prior to participating in the study. Participants provided consent for all aspects of the study including consent to participate in the study and consent to publish any findings from the study. Ethical approval was obtained from the Deakin University Human Research Ethics Committee (ref 2011–099). This committee is in compliance with the Helsinki Declaration.

### Participants

This study was comprised of a total of 101 participants. Fifty of these were adolescents with ASD with equal numbers of males and females (n = 25, respectively). The remaining 51 participants were TD adolescent males (n = 26) and females (n = 25). Participants were aged between 10 and 16 years (mean 12.87, standard deviation = 2.08). The age range, means and standard deviations for all participants are presented in Table 1. There were no significant differences in age between the groups by gender (*t*_(99)_ = 0.48, *P* > 05) or diagnosis (*t*_(99)_ = 0.69, *P* > 05).

**Table 1:** Mean ages and standard deviations for participants

	**Autism spectrum disorder**			**Typically developing**		
**Gender**	**n**	**Mean age**	**SD**	**n**	**Mean age**	**SD**
Male	25	13.88	1.86	26	12.16	1.79
Female	25	13.56	2.10	25	11.84	1.90
Total	50	13.73	1.97	51	12.00	1.84

Participants were approached through a variety of sources including ASD support groups and databases. All participants with ASD were required to have received a formal diagnosis of autism from a Psychologist, Paediatrician, or a Child Psychiatrist, and to have been assessed as being high functioning (that is, having an IQ score greater than 70 points). Although IQ scores were above 70 as assessed by a qualified Psychologist, Paediatrician, or a Child Psychiatrist, participants were not re-tested but were excluded if their reported IQ was below 70. Individuals were excluded from the study if they had a diagnosis of low functioning autism, or pervasive developmental disorder - not otherwise specified, or if they had a diagnosis of intellectual disability. In four cases, a co-morbid diagnosis of attention deficit hyperactivity disorder had also been received.

### Materials

Instruments were selected based on their appropriateness for children aged 10 to 16 years as well as for children with ASD. The scales were selected with the aim of examining the nature and understanding of friendships of the participants.

#### Friendship questionnaire

Originally developed by Baron-Cohen and Wheelwright [42], the FQ is designed to measure friendship quality, understanding and empathy. The FQ is a brief self-report measure that requires participants to respond to 35 items, 27 of which are scored as per the original scoring protocol [42]. The maximum score for each item is 5, with the scores ranging from 0 to 5. Five of the 35 items are negatively worded to assess response bias. The internal consistency of the measure is reportedly high at *a* = 0.84 [42]. As the FQ was originally designed for use with British adults, some rewording was deemed necessary for an Australian adolescent sample speaking an Australian idiom. The revised version was assessed by means of a pilot study using two Year 7 classes. The results from these pilot data informed further changes to items, resulting in 34 items, with 27 of these scored as per the instructions from Baron-Cohen and Wheelwright [40]. This revised version was delivered to participants in the form of a guided (semi-structured) interview, whereby the researcher reads the questions aloud and the child was allowed to follow the questionnaire with the researcher. A standard set of explanations was used if the child required further clarification (Additional file 1). The revised children’s version FQ demonstrated a weak internal consistency with *a* = 0.61 while the parent’s version of the FQ demonstrated a high internal consistency with *a* = 0.80.

#### Demographics questionnaire

A ten-item demographics questionnaire was completed by the parents to gather additional information, including the child’s age, gender, IQ, ASD diagnosis and other co-morbid diagnoses. The demographics questionnaire also gathered information about any potential ASD diagnoses in other family members.

### Procedure

Participants were tested at a location and time of their choosing, predominantly in private homes. Testing generally took 20 minutes for children and 30 minutes for their parents. Two researchers conducted every interview with the first sitting with the child to work through the structured interview. The second researcher sat with the parent who completed the same FQ in a self-report format, but also completed the demographic questionnaire. The parents were asked to complete the questionnaire about their child, focusing on what they believed to be the reality of the situation rather than how they thought the child would respond. Parents and children were tested in separate areas to prevent intentional or unintentional collusion.

## Results

The study used a 2 × 2 between-subjects design. The independent variables were diagnosis (ASD versus TD) and gender (female versus male). Data were screened for outliers and missing data. No outliers were identified and no data were missing. Data were also screened for normality. As recommended by Tabachnick and Fidell [44], normality was assessed separately within experimental conditions. No skew or kurtosis was identified in any of the variables. Normality was further assessed with the Kolmogrov-Smirnov test, which was assessed against a *P* value of 0.05. Violations of this criterion were not present for any of the variables indicating that normality can be assumed [45]. Homogeneity of variance was assessed for grouped data by inspection of Levene’s test. No significant differences were detected for gender or diagnosis which revealed that normality was met for those variables when compared to a *P* value of 0.01 as recommended by Tabachnick and Fidell [44].

A two-way random Intraclass Correlation Coefficient was conducted to assess the specific relationship between parent and child reports on the FQ. The two sources of reports demonstrated a significant correlation of r = 0.519, *P* < 0.05.When considering a single rater, a significant correlation of r = 0.351, *P* < 0.05, was found.

A two-way factorial analysis of variance was conducted to examine the main effect of gender on scores for the children’s version of the FQ. Gender was found to be a significant predictor of participants FQ scores (*F*_(1, 101)_ = 22.66, *P* < 0.001, η_p_^2^ = 0.20). The means and standard deviations displayed in Table 2 show that females scored higher than males on the FQ. Diagnosis was also found to be a significant predictor of the participants’ FQ scores (*F*_(1, 101)_ = 17.54, *P* < 0.001, η_p_^2^ = 0.16). Means and standard deviations displayed in Table 2 show that participants diagnosed with ASD scored lower than TD participants on the FQ, regardless of gender. The effect of diagnosis did not depend upon gender, insofar as children with ASD of both genders had a similar reduction in FQ scores (*F*_(1, 101)_ = 1.00, *P* > 0.05, η^2^ = 0.01).

**Table 2:** FQ scores for child by gender and diagnosis, total FQ scores for parents by child’s gender and diagnosis, and differences in scores between parent and child for FQ score by child’s gender and diagnosis

			**FQ score**	
**Child gender**	**Diagnosis**	**n**	**Mean**	**SD**
Children				
Male	ASD	25	61.48	15.64
	TD	26	74.76	12.15
	Total	51	68.25	15.37
Female	ASD	25	76.76	13.97
	TD	25	84.84	9.91
	Total	50	80.80	12.66
Total	ASD	50	69.12	15.58
	TD	51	79.70	2.12
	Total	101	74.45	15.38
Parents				
Male	ASD	25	43.04	19.25
	TD	26	68.58	11.19
	Total	51	56.06	20.16
Female	ASD	25	54.84	20.06
	TD	25	75.84	15.98
	Total	50	65.34	20.85
Total	ASD	50	48.94	20.35
	TD	51	72.14	14.10
	Total	101	60.65	20.93
**Diagnosis**	**Child gender**		**Mean**	**SD**
ASD	Male	25	−18.44	18.28
	Female	25	−21.92	21.45
	Total	50	−20.18	19.80
TD	Male	26	−6.19	17.34
	Female	25	−9.00	16.32
	Total	51	−7.57	16.74

ASD, autism spectrum disorder; FQ, friendship questionnaire; TD, typically developing.

A two-way factorial analysis of variance was conducted to examine the main effect of gender on scores for the parent’s version of the FQ. Gender was found to be a significant predictor of participants FQ scores (*F*_(1, 101)_ = 7.99, *P* < 0.01, η^2^ = 0.08). The means and standard deviations displayed in Table 2 show that parents of females scored higher than parents of males on the FQ. Diagnosis was also found to be a significant predictor of the participants’ FQ scores (*F*_(1, 101)_ = 47.67, *P* < 0.001, η^2^ = 0.33). Means and standard deviations displayed in Table 2 show that participants diagnosed with ASD scored lower than TD participants on the FQ, regardless of gender. The effect of diagnosis did not depend upon gender, insofar as parent reports of children with ASD of both genders had a similar reduction in FQ scores (*F*_(1, 101)_ = 0.453, *P* < 0.05, η^2^ = 0.005).

To test the hypothesis that there would be a significant difference between the FQ scores of parents and their children, an independent samples *t*-test was conducted. Average differences of the mean total scores and the standard deviations for parents and children were calculated. It was predicted that children would consistently overestimate their scores on the FQ compared to their parents, and this was supported (*t*_(100)_ = 5.43, *P* < 0.001). Additional analysis revealed a significant difference also existed between TD children and their parents’ perceptions of friendships (*t*_(50)_ = 2.91, *P* < 0.005, *d* = 0.58). Parents of both cohorts reported lower mean FQ scores, as indicated by the negative mean difference for FQ scores in Table 2.

**Figure 1:**
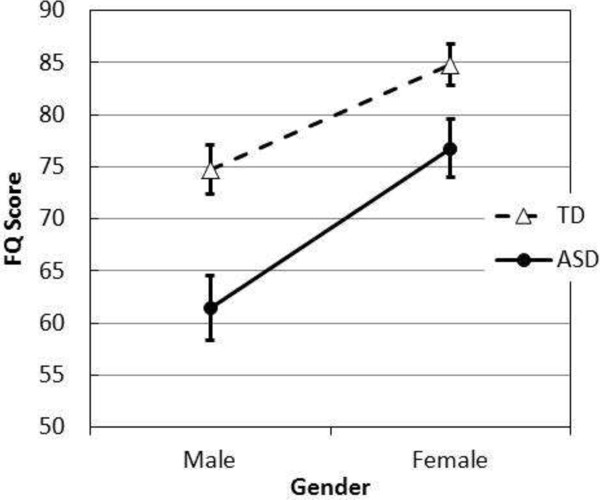
**Total friendship questionnaire (FQ) scores by gender and diagnosis.** Error bars represent standard errors of the mean. ASD, autism spectrum disorder; TD, typically developing.

After an examination of the graph (Figure 1) and means, an independent samples *t*-test was conducted to examine the similar mean FQ scores between females with ASD and TD males. No significant differences were found (*t*_*(*100)_ = −2.36, *P* > 0.05), indicating that females with ASD demonstrated similar scores on the FQ to TD males (Table 2). This pattern of scores is represented in Figure 1.

An independent samples *t*-test was conducted to examine the mean FQ scores between females with ASD and males with ASD. A significant difference was found across gender (*t*_(48)_ = −3.64, *P* < 0.05). Examination of Table 2 reveals males with ASD displayed much lower scores on the FQ when compared to females with ASD.

## Discussion

This study investigated the female phenotype of ASD by examining gender differences in friendship quality for children with ASD. It was predicted that females with ASD would score higher than males with ASD on the FQ. It was also expected that, regardless of diagnosis, female participants would score higher than male participants. As it has been well established that people with ASD experience social deficits, it was also anticipated that TD participants would score higher than ASD participants, regardless of gender.

Overall, the findings support the notion that important differences exist between males and females, and examination of Figure 1 reveals that persons with ASD were consistently lower than their TD counterparts. Combined with a significant effect for diagnosis, this suggests some parallelism between groups across gender, where children with ASD are consistently lower and, thus, girls with ASD appear consistently lower. Unlike Baron-Cohen and Wheelwright [42], it was found that females with ASD scored significantly higher on the FQ than males with ASD. This finding is consistent between reports from both children and their parents. This result is interesting in that it supports clinically anecdotal evidence that females with ASD develop a capacity to camouflage or hide their social insecurities in order to fit in [9,35-38,46]. As to why Baron-Cohen and Wheelwright did not find a similar result to that reported here, it is highly likely that Baron-Cohen and Wheelwright’s within-group variability was dramatically increased by the wide age ranges they used (14 to 64 years) which, in turn, would be more likely to mathematically overwhelm the between-group differences, rendering the ability to find a significant result much less probable.

Consistent with predictions, it was also found that female participants scored significantly higher than male participants on the FQ, regardless of diagnosis, whereby higher FQ scores indicate higher levels of sociability, emotionality and friendship. This finding was consistent between both the child and parent ratings on the FQ. This prediction is in agreement with the literature, which suggests that females and women in general demonstrate greater ability to empathise, are more likely to be caretakers, and generate and maintain intimate relationships more readily than boys and men [15,23,47-49]. It was also found that, regardless of gender, children with ASD obtained lower scores on the FQ than TD children. This finding was concordant with the diagnostic criteria for ASD which requires individuals with ASD to demonstrate persistent difficulties with developing and maintaining appropriate, reciprocal friendships [50]. These findings extend those of Lai and colleagues [40] and Dworzynski and colleagues [6] who also identified that both adolescent and adult females with ASD demonstrate fewer socio-communicative symptoms compared to their male equivalents.

Perhaps the most interesting, yet unexpected, finding was that females with ASD demonstrated similar FQ scores as TD males. Not surprisingly, should this result be replicated, it would go a considerable way to explaining the camouflage effect Wing proposed as obscuring the ready diagnosis of young women [39]. Unfortunately, current diagnostic criteria do not differentiate between the social presentation of males and females with ASD [50]. This finding suggests that, while females with ASD do not demonstrate the same score on the FQ as TD females, their levels might superficially appear normal, being similar to that of TD males, and considerably higher than males with ASD. Others have previously reported this in the clinical literature. Attwood [35] describes the apparent social function of females with ASD to be closer to that of TD females than males with ASD whereby females with ASD demonstrate a superficial understanding of appropriate social etiquette, and are able to mask their deficits through imitation and mimicry. Attwood’s clinical description and this result both support Wing’s Camouflage Hypothesis [39] which suggests that females with ASD are able to mask or camouflage their social deficits. In addition, supporting Wing’s Camouflage Hypothesis, the findings may also support Baron-Cohen’s Extreme Male Brain theory with females with ASD presenting with TD male social characteristics.

This apparent advantage that females with ASD demonstrate over males with ASD may partially account for the skewed gender ratio within ASD. Were diagnostic criteria on the social-communication domain to consider gender differences, perhaps a larger number of females might be diagnosed. A naïve clinician may, considering a female presenting for an ASD diagnosis, incorrectly assume that the individual is not on the spectrum because she manifests social skills at a level similar to TD boys. Naturally, such missed diagnoses place girls on the spectrum at a gender-based disadvantage by not being given fair and equal access to adequate intervention.

Importantly, the pattern of findings between parent and child report was the same. Specifically, both cohorts identified significant effects of gender and diagnosis while there was no interaction effects found between these variables. This pattern is not surprising as the nature of autism presupposes deficits in social understanding, as measured by the FQ. In relation to the congruent finding of gender differences, this outcome is encouraging as it further supports the hypothesis that females with ASD demonstrate presentation differences compared to males with ASD.

As expected, there was a significant difference between the scores produced by the children and their parents. Parents consistently produced lower scores on the FQ than their children, across all groups. The greatest disparity was detected between females with ASD and their parents. It is possible that the females with ASD develop an intellectual understanding of the rules associated with sociability, emotionality and friendship whereby they then translate this understanding to their answers on the FQ. Given that this study did not incorporate any observational components, it was unclear whether these females put their apparent understanding into real world action. However, if parental responses are also considered in this context, it becomes apparent that, in practice, females with ASD do not always follow through in social situations. It is possible that there is parent bias in reporting, where parents perceive the social challenges of girls as being more severe than boys. On the other hand, although it is possible that the parents’ responses are not representative of their children, it is more probable that their responses represent the reality of their children’s behaviour. Additionally, parents were instructed to respond to the items based on what they believe is the reality of their children’s behaviour, not how they believed their child would respond. This difference may also account for the apparent differences in scores between the parents and their children.

A greater disparity was observed between the children with ASD and their parents than TD children and their parents, as predicted. Given that it is well established that children with ASD often lack insight [9] it is possible that the parents' reports were more realistic, but this warrants further investigation. There was a relatively low concordance between the parent and children reports (27% agreement between the reports). This finding is not surprising, however, given that individuals with ASD often demonstrate difficulties with insight into their own behaviours and relationships [51,52]. Moreover, female children (regardless of diagnosis) demonstrated a greater gap between their scores and those provided by their parents than male children. Underlying reasons for this difference remain unclear and further testing of these phenomena is needed.

It is currently estimated that four males are diagnosed with ASD for every one female [53], leading to the natural conclusion that ASD is rarely identified in females [53]. According to Dworzynski and colleagues [6], gender ratios begin to even out in lower functioning individuals, or are less apparent. This suggests that, in those individuals who are higher functioning, there may be a high proportion of females who ‘pass’ under the radar, raising the added complexity of determining who should receive a diagnosis. The focus of this study was on the social domain; however, it is important to also examine symptom severity between genders in other domains. If these were more severe in high functioning females than males, this would be consistent with the idea that high functioning ASD females may go unnoticed because of stronger social skills, and thus require more severe symptomatology in other areas to obtain a diagnosis. Clinical accounts have suggested ASD is not simply male-centric, but rather it is the diagnostic systems which are biased towards the identification of males with ASD [9]. It is believed that the underlying cause of this situation is a self-fulfilling research process, where the majority of study participants have been males with ASD. This has led to the development of male-focussed diagnostic criteria that (to a certain degree) exclude females from attaining a diagnosis of ASD.

As previously discussed, the current diagnostic criteria for ASD do not take into account universally accepted gender differences in sociability, friendships and emotionality. These differences challenge the notion that individuals with ASD, regardless of gender, present in a similar way, at least behaviourally. Instead, it was expected in this study that females with ASD would demonstrate higher scores on the FQ when compared with males with ASD. The new Diagnostic and Statistical Manual of Mental Disorders Fifth Edition has noted that gender differences probably exist on the ASD spectrum, but these differences have not yet been incorporated into the diagnostic criteria.

### Limitations and future directions

The study had a number of limitations. While all high functioning autism participants had previously received a diagnosis from a clinical psychologist, diagnoses were not directly confirmed using the Autism Diagnostic Observation Schedule or Autism Diagnostic Interview Raised. As such, it is possible that some of the diagnoses may have been made in error or out of date and could therefore have confounded the findings. Future studies of this nature should undertake measures to confirm diagnosis. Additionally, the FQ contained items such as telephone use that were not necessarily appropriate to the sample used and, although it was modified, that modification itself had not been validated and the relatively low reliability of this measure may have, again, confounded the impact on results. The low internal consistency evident in the revised FQ strongly suggests the need for further testing of this instrument. Lastly, some observational confirmation of the measures would have greatly improved the study.

### Implications

These findings demonstrate that, while females with ASD do not display FQ scores at levels expected for TD females, they do have superior sociability skills compared to their ASD male counterparts. Thus, these results suggest some further reconceptualisation of the current diagnostic criteria for ASD, considering the differential effect of social skills apparent in females with ASD when compared to males with ASD. In addition, these findings suggest the need for social intervention that is specifically tailored for females with ASD based on the fact that their social capacities are qualitatively different to those of males with ASD.

## Conclusions

This study aimed to explore the disparate gender ratio within ASD. Overall, female participants were found to demonstrate higher levels on the FQ when compared to male participants. This difference was also seen within the ASD cohort, whereby females with ASD demonstrated higher levels on the FQ than males with ASD. In general, children with ASD demonstrated lower levels on the FQ when compared to their TD counterparts. Further, parents of children with ASD generated lower scores than their children with the greatest difference observed between females with ASD and their parents.

What was unexpected was the finding that TD males and females with ASD exhibited similar FQ scores, supporting the notion that there may be a distinct female profile of ASD. This finding is supported by clinical anecdotes reporting that females with ASD have highly developed facilities for mimicry and imitation, as well as more highly developed social skills than would be expected. Further research is now required to examine the underlying causes for this phenomenon. For example, it is possible that females with ASD demonstrate relative strengths in socio-communicative abilities compared to males with ASD. Further examination of this phenomenon is necessary in order to develop gender-appropriate diagnostic criteria and gender-appropriate interventions for ASD.

## Abbreviations

ASD: autism spectrum disorder; FQ: friendship questionnaire; IQ: intelligence quotient; TD: typically developing.

## Competing interests

The authors declare that they have no competing interests.

## Authors’ contributions

AMH conceived the study, and participated in its design and coordination, carried out the data collection, data analysis and drafted the manuscript. JAM and MAS helped to draft the manuscript. All authors read and approved the final manuscript.

## Supplementary Material

Additional file 1 The Friendship Questionnaire. Click here for file
