# Supplementary material for: Gender differences in emotionality and sociability in children with autism spectrum disorders
Source: Mol Autism. 2014 Feb 28;5:19. doi: 10.1186/2040-2392-5-19 (PMC3945617; doi:10.1186/2040-2392-5-19)
Supplement: Additional file 1 — The Friendship Questionnaire. [file 2040-2392-5-19-S1.doc]

**Additional file 1**

**The Friendship Questionnaire (FQ)**

**(Text in bold indicates standardised explanatory terms used for clarification of questions when needed)**

This questionnaire has 35 questions. Please answer every question.

For each of the following questions, tick the box next to the statement which most applies to you.

1.a I have one or two best friends.

a.b I have a couple of friends who I call best friends.

b.c I don’t have anybody who I would call a best friend.

2. The most important thing about a friendship is:

a having someone to share secrets with.

b having somebody to have fun with.

3. If I had to pick, I would rather have a friend who:

a enjoys doing the same things as me

b feels the same way about **things** as I do

4. Which of the following best represents you?

a I like to be close friends with people.

b I don’t like to be close friends with people.

5. When I talk with friends on the phone, it is usually to:

a make plans rather than to chat.

b chat rather than to make plans.

***Plans: going to movies, coming over to hang out.***

6. Which of the following best represents you?

a I normally think of an activity I want to do and then find somebody to do it with.

b I normally plan to meet somebody and then think of something to do.

***Activity: going to movies, coming over to hang out.***

7. Which of the following best represents you?

a I like to meet a friend for an activity, like, going to a friends house or the movies.

b I like to meet a friend for a chat, like, at their house or at a cafe.

***Chat = talk***

8. If I moved to a new area, I would try harder to:

a see old friends more often.

b make new friends

9. My friends see me as someone who is:

a more caring than fun

b more fun than caring

***Explain: Dichotomy, not just caring or fun. Can be both, but which one MORE.***

10. If a friend had a problem, I would be better at:

a talking about their feelings

b helping them figure out what to do

11. If a friend was having problems, I would:

a wait for them to talk to me as I wouldn’t want to butt in or be nosey.

b ask them about it.

***Problem: upset about something***

12. When I have a problem, I think that it is better to:

a work it out on my own.

b talk about it with a friend.

c try and forget about it.

13. If I have to say something that might sound mean to a friend, I think it’s best to:

a choose my words carefully.

b just come right out and say it.

***Choose words: be careful about how I say it.***

14. If I had a fight with a good friend and I thought that it was their fault, I would

a do whatever it takes to fix the friendship.

b be the first one to try to fix it, as long as they tried as well.

c be happy to fix the problem, if they tried first.

d not be good friends with them anymore.

15. Usually, my favorite place to hang out would be

a in my room, by myself.

b in my room, with friends coming over sometimes.

c in my room with one or two friends.

d in the family room.

**For the next set of questions, please circle the option to indicate your answer.**

16. How easy do you find it to talk about your feelings with your friends?

Very easy

Easy

Not very easy

Difficult

Very difficult

17. How easy would you find it to talk about your feelings with someone I don’t know?

Very easy

Easy

Not very easy

Difficult

Very difficult

18. How similar is your personality to your friends?

Very similar

Similar

Not very similar

Very dissimilar

**Personality: What makes you you.**

19. How much do your friends like to do the same things as you?

A lot

Not much

20. How important is it to you what your friends think of you?

Not important

Very important

21. How important is it to you what people you don’t know think of you?

Not important

Very important

22. How easy is it to tell your friends that you’re wrong?

Very easy

Easy

Not very easy

Difficult

Very difficult

**Preamble: I’m not very good at running. And I don’t think that I’m very good at Maths either. Is there something that you’re not very good at?**

23. How easy would it be to tell a friend that you’re not very good at….?

1: Easy to 10: Difficult

24. How easy do you find it to tell a friend about the things I do well?

Very easy

Easy

Not very easy

Difficult

Very difficult

25. How interested are you in the everyday details (e.g., their relationships, family, what’s currently going on in their lives) of your friends’ lives?

Completely disinterested

Not very interested

Quite interested

Very interested

26. When you are in a group of friends, is it important you to know the “gossip,”? e.g., who **dislikes** who, who’s had a relationship with who, secrets.

Of no importance

Of little importance

Fairly important

Very important

Of great importance

***Gossip: what’s going on in the group***

27. Which is more important:

School work

Friends

Both are equal

28. How often do you meet up with friends?

Once or twice a year

Once every 2 or 3 months

Once a month

Once every couple of weeks

Once or twice a week

3 or 4 times a week

More than any of the above

29. How would you prefer to keep in touch with friends?

(Please put: 1 in the box next to your most preferred **method** 2 in the box next to your second **preference** 3 in the box next to your third **preference**)

Social networking (eg, Facebook, Club Penguin, MSN)

Texting

Email

Phone calls

Face-to-face

30. How easy to do you find it to make new friends?

Very easy

Easy

Not very easy

Difficult

Very difficult

31. How much do you need to be around people in a day?

No contact—I don’t get lonely

Just being near to people, even if I am not talking to them

A casual chat, e.g., with a teacher or canteen lady.

A chat with a friend

Two or three chats with friends during the day

More than any of the above

32. How much do you need to be around people in a week?

None—I don’t get lonely

Being around people, even if I wasn’t talking to them

Casual chats, e.g., with a teacher or canteen lady

One chat with a friend

Two or three chats during the week with friends

One chat every day with a friend

Two or three chats every day with a friend

More than any of the above

33. When talking with friends, how much time do you spend talking about the following:

**About the following: *(Please put: 1 in the box next to the topic that you talk most about, 2 in the box next to the topic you talk next most about, etc., through to7 in the box next to the topic you talk least about. Use each number only once, i.e., there should be no ties) (same, repeats.)***

Things on the news

Hobbies and interests (e.g., sport, TV, music, movies, fashion, holidays, horse riding, drawing, etc.)

Personal things (e.g., fights, feelings etc.)

School or work

Family and friends

The weather

What you’ve been doing since last time you spoke

34. When you meet someone for the first time, how likely are you to talk

**About the following: *(Please put: 1 in the box next to the topic* *that you talk most about,* *2 in the box next to the topic* *you talk next most about,* *etc., through to7 in the box next to the topic* *you talk least about.* *Use each number only once, i.e., there should* *be no ties.)***

Things on the news

Hobbies and interests

Personal matters

School or work

Family and friends

The weather

What you’ve been doing recently

***Thank you for completing this questionnaire.(quiz)***
